# Probiotics in the Management of Chronic Bacterial Prostatitis Patients: A Randomized, Double-Blind Trial to Evaluate a Possible Link Between Gut Microbiota Restoring and Symptom Relief

**DOI:** 10.3390/microorganisms13010130

**Published:** 2025-01-10

**Authors:** Cristina Vocca, Diana Marisol Abrego-Guandique, Erika Cione, Vincenzo Rania, Gianmarco Marcianò, Caterina Palleria, Luca Catarisano, Manuela Colosimo, Gregorio La Cava, Italo Michele Palumbo, Giovambattista De Sarro, Tommaso Ceccato, Simone Botti, Tommaso Cai, Alessandro Palmieri, Luca Gallelli

**Affiliations:** 1 Operative Unit of Clinical Pharmacology and Pharmacovigilance, Department of Health Science, AOU Dulbecco, University Magna Graecia of Catanzaro, 88100 Catanzaro, Italy; cristina_vocca@live.it (C.V.); dianamarisol.abregoguandique@unicz.it (D.M.A.-G.); raniavincenzo1@gmail.com (V.R.); gianmarco.marciano3@gmail.com (G.M.); palleria@unicz.it (C.P.); lucacatarisano@gmail.com (L.C.); desarro@unicz.it (G.D.S.); gallelli@unicz.it (L.G.); 2 Department of Pharmacy, Health and Nutritional Sciences, University of Calabria, 87036 Rende, Italy; erika.cione@unical.it; 3 Operative Unit of Microbiology and Virology, AOU Dulbecco, 88100 Catanzaro, Italy; manuelacolosimo@hotmail.it; 4 Urology Division Azienda Sanitaria Provinciale, Department of Primary Care, 88100 Catanzaro, Italy; gregoriolacava@libero.it; 5 Department of Urology, University Magna Graecia of Catanzaro, 88100 Catanzaro, Italy; itapal73@gmail.com; 6 Research Center FAS@UMG, Department of Health Science, University Magna Graecia of Catanzaro, 88100 Catanzaro, Italy; 7 Department of Urology, Santa Chiara Regional Hospital, 38123 Trento, Italy; tommaso.ceccato@apss.tn.it (T.C.); simone.botti@apss.tn.it (S.B.); 8 Institute of Clinical Medicine, University of Oslo, 0313 Oslo, Norway; 9 Department of Urology, Federico II University of Naples, 80138 Naples, Italy; info@alessandropalmieri.it; 10 Medifarmagen, University of Catanzaro and Renato Dulbecco Hospital, 88100 Catanzaro, Italy

**Keywords:** microbiota, chronic bacterial prostatitis, antibiotic resistance, *Lactobacillus casei* DG, probiotics

## Abstract

Several studies have suggested that probiotics could play a role in the management of patients with chronic bacterial prostatitis (CBP). In this randomized, placebo-controlled clinical study, we evaluated the efficacy and safety of consumption of probiotics containing human *Lactobacillus casei* DG^®^ as an add-on treatment in patients with clinical recurrences of CBP, through gut microbiota modification analysis. Enrolled patients with CBP were randomized to receive for 3 months probiotics containing human *Lactobacillus casei* DG^®^ or placebo following 1 month treatment with ciprofloxacin. During the enrollment and follow-ups, urological examinations analyzed symptoms and quality of life, while microbiological tests analyzed gut and seminal microbiota. During the study, the development of adverse drug reactions was evaluated through the Naranjo scale. Twenty-four patients with CBP were recruited and treated for 3 months with placebo (n. 12) or with *Lactobacillus casei* DG^®^ (n. 12). *Lactobacillus casei* DG^®^ induced a significantly (*p* < 0.01) faster recovery of symptoms than placebo (2 days vs. 8 days) and an increased time free from symptoms (86 days vs. 42 days) without the occurrence of adverse events. In the probiotic group, the appearance of *Lactobacilli* after 30 days (T1) was higher vs. the placebo group, and a significant reduction in *Corynebacterium*, *Peptoniphilus*, *Pseudomonas*, *Veillonella*, *Staphylococcus,* and *Streptococcus* was also observed. These preliminary data suggest that in patients with CBP, the use of *Lactobacillus casei* DG after an antimicrobial treatment improves the days free of symptoms and the quality of life, without the development of adverse drug reactions.

## 1. Introduction

Chronic prostatitis is a chronic inflammation of the prostate gland affecting men of all ages with a prevalence of 14% under 50 years of age; of these, 10% would be of bacterial origin [1,2]. According to the National Institutes of Health (NIH), 7–14% of all cases are chronic bacterial prostatitis (CBP), which is classified as category II and impairs the quality of life [3,4,5]. Usually the treatment of CBP involves the use of fluoroquinolones for a long time, with an increased risk of antibiotic resistance and interactions in poly-treated patients [6]. The pathophysiology of CBP appears related to both bacterial biofilm [3] and prostate inflammation [7], suggesting that bacterial infections contribute to the prostatic inflammatory response. Bacterial intestinal reservoirs may be linked to these infections by increasing the permeability of the intestinal mucosa.

Several studies reported that probiotics, particularly lactobacillus, have local anti-inflammatory effects by modulating the intestinal flora [8].

Borruel et al. [9] in an ex vivo study performed on intestinal tissues explanted from patients with Crohn’s disease, documented that *Lactobacillus casei* and *Lactobacillus bulgaricus* were able to downregulate the production of proinflammatory cytokines.

The effects of probiotics on bacterial intestinal growth could be related to the enhancement of intestinal barrier function [10,11] as well as to their immune modulatory properties [12,13], though an ability to produce antimicrobial peptides has been postulated [14].

An interesting study suggested that *Lactobacillus casei* modulated the expression of several proinflammatory mediators [15]. Moreover, an experimental animal study reported that oral administration of *Lactobacillus casei* was able to reduce the serum level of Interleukin-6 (IL-6) and 10 and tumor necrosis factor-α [16].

Other studies have suggested that probiotics may play a part in prostatic illnesses by modulating the inflammatory pathway that controls the inflammatory condition of the intestine [17,18,19].

Consistent with this, recent studies hypothesized that *Lactobacillus* strains in association with antibiotic treatment might both reduce the development of adverse drug reactions and improve the quality of life of patients with prostate diseases, probably through change in the gut microbiota [19,20,21].

However, up to this moment, no demonstration of the role of probiotics in modifying the composition of gut microbiota and its impact on CBP has been reported. This study aims to evaluate the clinical efficacy and safety of probiotics in patients with chronic bacterial prostatitis already treated with fluoroquinolones by analyzing the symptomatology, the frequency of relapses, and the changes in the composition of the gut microbiota.

## 2. Materials and Methods

### 2.1. Study Design

This is a randomized, double-blind, placebo-controlled, multicenter clinical trial examining the efficacy and safety of consumption of probiotics containing human *Lactobacillus casei* DG^®^ vs. placebo, both following treatment with ciprofloxacin in patients with chronic bacterial prostatitis. *Lactobacillus casei* DG^®^ is defined as probiotic, in line with the FAO/WHO definition—“live microorganisms which when administered in adequate amounts confer a health benefit on the host” [22]. The study was conducted by the Pharmacology and Pharmacovigilance Unit of the Magna Graecia University of Catanzaro, Dulbecco University Hospital of Catanzaro, Urology Unit of the Territorial Health Department of the Catanzaro and Urology Unit of Dulbecco University Hospital of Catanzaro from March 2022 to April 2024. During the intervention period, the enrolled participants consumed their assigned products twice a day for 3 months.

Patients were enrolled at baseline (T0) and then were monitored at 30 days (T1), 90 days (T2), and 180 days (T3) to evaluate adherence to treatment. At the beginning of the study, participants were asked not to change their usual dietary habits during treatment.

All methods of collecting biological samples from stools used under this medical examination were taken with the approval of the attending physician. The study was conducted in accordance with the guidelines of the 1964 Declaration of Helsinki and its later amendments. All patients or their guardians signed a written informed consent. This study was approved by the Local Ethical Committee of Calabria Centro (ID number 258 of 19 September 2019).

### 2.2. Participants

In this study, 24 patients were enrolled from the Urology Unit of the Territorial Health Department of the Catanzaro and Urology Unit of Dulbecco University Hospital of Catanzaro, according to the following eligibility criteria:

(1) Subjects between 18 and 55 years old with clinical, instrumental, and microbiological diagnosis of chronic prostatitis, according to indications of the EAU and history of symptomatology related to the diagnosis of chronic bacterial prostatitis for more than 6 months [23,24].
(2) Isolation of uropathogens, according to EAU indications, in microbiological samples from Meares–Stamy tests or semen culture [25].
(3) NIH-CPSI > 9 and change in IPSS and SF-36 questionnaires (no significant cut-off is indicated), in line with EAU recommendations [26].
(4) Subjects who can follow the study and give their consent to enroll in the study.

Patients with established organic bowel disease (including celiac disease or inflammatory bowel disease), allergies related to fluoroquinolones, infectious intestinal diseases, psychiatric illnesses and/or psychological disorders, active malignancy of any type or history of malignancy (patients with a history of other malignancies that have been surgically removed and who have no evidence of relapses for at least five years prior to study enrollment are also acceptable), were excluded. In the investigator’s judgment, patients with a Charlson Comorbidity Index >2 or with any severe pathology that may interfere with study treatment were also excluded. We also excluded subjects who had undergone prior antibiotic therapy (for any indication) within the last 3 months or who were on current antibiotic therapy, and those with chronic use of probiotics or supplements/herbal remedies in the month before the start of the trial, or who had undergone previous abdominal surgeries. Also considered as exclusion criteria were episodes of viral or bacterial enteritis within 2 months prior to the study, recent history or suspicion of alcohol or drug abuse, inadequate reliability or presence of conditions that may lead to patient non-compliance/adherence to the protocol, or prior participation in this study or concurrently with other clinical trials.

### 2.3. Drugs

During the study, patients were treated for 3 months with

- 1 capsule/day containing 24 billion live cells of *Lactobacillus casei* DG^®^ (Enterolactis^®^ plus), supported by Alfasigma S.p.A.—Via Ragazzi del ‘99, 5—Bologna (Italy).
- 1 capsule/day in packaging identical to probiotic with the same color, weight, smell and taste, but without bacteria, supported by Alfasigma S.p.A.—Via Ragazzi del ‘99, 5—Bologna (Italy).

### 2.4. Experimental Protocol

Enrolled patients were randomized, using a 1:1 protocol of randomization, into two groups: Group A: probiotic (n: 12). and Group B: placebo (n: 12). The subjects were identified with a numerical code to preserve privacy. Before the beginning of the study, and during follow-up(s), urological evaluation was performed, and urologists administered questionnaires. Clinical and laboratory data were collected directly by the medical staff involved in the study.

### 2.5. Questionnaires

Patients were administered the following questionnaires:

(1) International Prostatic Symptoms Score (IPSS) consisting of 8 questions used to screen, diagnose, and monitor symptoms linked to benign prostatic hyperplasia. The answers are given points on a scale of 0 to 5. The IPSS values were classified as mild (scores 0–7), moderate (scores 8–19), and severe (scores 20–35) non-neurologic lower urinary tract symptoms, specifically, incomplete bladder emptying, frequency, intermittency, urgency, weak stream, straining to void, and nocturia [27].
(2) NIH-Chronic Prostatitis Symptom Index (NIH-CPSI), consisting of 9 items and used to assess the symptom severity of prostatitis and the effectiveness of treatment. NIH-CPSI includes three subscales with a total score ranging from 0 to 43: pain or discomfort (4 items with a total score ranging from 0 to 21), urinary symptoms (2 items with a total score ranging from 0 to 10), impact on the quality of life (3 items with a total score ranging from 0 to 12 points). The scores are higher as the symptoms become more severe [28].
(3) International Index of Erectile Function (IIEF-5), consisting of 5 questions indicating the presence of erectile dysfunction, each of which can be scored from 0 or 1 (representing the worst) to 5 (representing the best). The final score ranges from 1 to 25 points. Erectile dysfunction is classified based on the overall score as severe (score 0 to 7), moderate to severe (score 8 to 11), mild to moderate (score 12 to 16), mild (score 17 to 21), and absent (score 22 to 25) [29].
(4) The 36-Item Short-Form Health Survey (SF-36), consisting of 36 questions and used to assess quality of life about pathology and the effectiveness of treatment. Quality of life is defined as the subjective perception of one’s own well-being within a socio-cultural context or as the satisfaction of desires and pleasures. Questions are summarized in two component summary scores, the Physical Component Summary (PCS) and the Mental Component Summary (MCS), representing eight concepts of health: physical functioning (PF), bodily pain (BP), role limitations due to physical health problems (RP), role limitations due to personal or emotional problems (RE), general mental health (MH), social functioning (SF), energy/fatigue or vitality (VIT), and general health perceptions (GH). A higher score represents better health, while a low score corresponds to a lower quality of life [30,31].
(5) Zung’s Self-Rating Anxiety Scale (Zung SAS) consists of a 20-item question scale that rates the four common characteristics of anxiety, both psychological and somatic. Responses are given on a 4-point scale, which range from 1 (none or a little of the time) to 4 (most or all of the time). Items include both negative and positive experiences. The final score ranges from 20 to 80 points. Anxiety is classified as normal (score 0 to 44), moderate (score 45 to 59), and severe (score 60 to 80) [32].
(6) Zung’s Self-Rating Depression Scale (Zung SDS) consists of a 20-item question scale that rates the four common characteristics of depression. Items tap psychological and physiological symptoms: 10 express negative experiences and 10 express positive experiences. Responses are given on a 4-point scale ranging from 1 (none or a little of the time) to 4 (most or all of the time). Total raw scores range from 20 to 80. Depression is classified as normal (score 20 to 49), mild (score 50 to 59), moderate (score 60 to 69), and severe (score 70 to 80) [33].

### 2.6. Meares–Stamey Test

Patients enrolled in the study underwent a thorough microbiological evaluation with the Meares–Stamey test to identify bacteria and leukocyturia in three/four biological samples: first void urine (VB1), second void urine (VB2), prostate massage secretion (EPS), post-massage urine (VB3) [34].

### 2.7. Fecal Samples

The samples were kept at ambient temperature until analysis, which was usually performed within 4 to 5 h of receipt. Analysis was performed 7 days a week, and then samples were tested within 24 h of collection.

### 2.8. Microbiological Identification Tests

The gut microbiota was analyzed using both QIAstat-Dx^®^ Gastrointestinal Panel (QIAGEN S.r.l., Milano, Italy) and culture isolation tests. Analysis with QIAstat-Dx^®^ Gastrointestinal Panel 1 was used according to the manufacturer’s instructions. The analysis requires approximately 50–200 mg of feces collected with a flocked swab from the FaecalSwab sample collection system (Copan, Brescia, Italy) that is resuspended in 2 mL of CaryBlair transport medium. A total of 200 μL of the FaecalSwab suspension was collected using a transfer pipette and loaded into the liquid sample port of a QIAstat-Dx^®^ Gastrointestinal Panel cartridge. All reactions are performed by the closed QIAstat-Dx^®^ system within the loaded cartridge and include lysis, extraction, amplification, and measurements of fluorescence of the amplified PCR products. The QIAstat-Dx^®^ Analyzer Software 1.6 interprets the results and generates test reports used to evaluate potential gastrointestinal pathogen findings. An internal control is included in the assay to monitor the quality of the reactions for a given sample. If the internal control is reported positive, all results are valid. If the internal control is reported negative, only positive results for targets are valid, while negative results for targets are invalid. Running a sample with the QIAstat-Dx^®^ Gastrointestinal Panel takes approximately 70 min/sample. Consistent with its manufacture, the QIAstat-Dx^®^ Gastrointestinal Panel detects the following pathogens: *Campylobacter* spp. (*C. jejuni*, *C. upsaliensis*, and *C. coli*), Clostridioides difficile tcdA/tcdB, enteroaggregative *E. coli* (EAEC), enteropathogenic *E. coli* (EPEC), enterotoxigenic *E. coli* (ETEC) eltA/estA, Shiga toxin–producing *E. coli* (STEC) stx1/stx2, Shiga toxin–producing E. coli (STEC) stx1/stx2 O157, enteroinvasive E. coli (EIEC)/Shigella (*S. sonnei*, *S. fexneri*, *S. boydii*, and *S. dysenteriae*), *Plesiomonas shigelloides*, *Salmonella* spp., *Vibrio cholerae*, *Vibrio parahaemolyticus*, *Vibrio vulnifcus*, *Yersinia enterocolitica*, *Cyclospora cayetanensis*, *Cryptosporidium* spp. (*C. parvum*, *C. hominis*, *C. felis*, and *C. meleagridis*), *Entamoeba histolytica*, *Giardia lamblia*, adenovirus F40/F41, norovirus GI and GII, rotavirus, and sapovirus (I, II, IV, and V).

Microbiological tests and phase-contrast microscopy (Phase contrast 2, Nikon, Tokyo, Japan) were used to evaluate the other bacteria. The microbiological tests included Gram staining, evaluation of catalase, oxidase, urease, methyl red, citrate, triple sugar iron, gas formation, and fermentation for the identification of lactic acid bacteria isolates. The results were interpreted according to Bergey’s manuals [35]. The microbial composition of the samples was analyzed using the VSEARCH software (version v2.22.1) consistent with ref. [36].

### 2.9. Admission (T0)

All patients underwent urological and rectal examinations during the admission, and IPSS, NIH-CPSI, IIEF-5, Zung SAS, Zung SDS, and SF-36 questionnaires were administered. Following international guidelines, subjects who met the inclusion criteria were enrolled and treated with Ciprofloxacin 1000 mg daily for 4 weeks. At the end of antibiotic therapy, all patients underwent microbiological evaluation with the Meares–Stamey test, and a stool sample was collected from each patient to analyze the gut and seminal microbiota. Afterward, subjects were randomized into either the placebo group or the probiotic group.

### 2.10. Follow-Up Visits (T1, T2, T3)

Starting at T0, 30 days (T1), 90 days (T2), and the end of the study at 180 days (T3) were considered as follow-ups. At each follow-up, patients underwent urological examination and rectal examination, and each was administered the IPSS, NIH-CPSI, IIEF-5, Zung SAS, Zung SDS, and SF-36 questionnaires. In addition, microbiological evaluation with the Meares–Stamey test, and/or semen culture, were performed and a stool sample was collected to assess any changes in the gut and seminal microbiota associated with probiotic intake. The dedicated database evaluated and recorded any systemic or local side-effects.

### 2.11. Outcomes

The primary outcomes included a statistically significant reduction (*p* < 0.05) in the number of prostatitis exacerbations in the human *Lactobacillus Casei* DG^®^ group vs. the placebo group, both taken from the end of antibiotic therapy. Instead, the second outcome included evaluation of the different evolutions of gut microbiota in the human *Lactobacillus casei* DG^®^ group vs. the placebo group, evaluation in the development of adverse events (assessed with the Naranjo Scale), reduction in recovery time of the disease relapses, and reduction in the intensity of prostatitis symptoms.

### 2.12. Statistical Analysis

The inter-group differences were evaluated by parametric methods (*t*-test). Correlation quotients were assessed using the Pearson correlation test. Normality was evaluated using the Shapiro–Wilk test, while ANOVA test and *t*-test were used to analyze the differences between the groups. Tukey and Benjamini–Hochberg tests were used to control type I errors. For all comparisons, differences were considered significant for *p* < 0.01. The *t*-test power was 0.6503918, and the Pearson correlation test power was 0.3136721.

## 3. Results

### 3.1. Population

During the study, 82 patients with chronic bacterial prostatitis were evaluated, and 24 patients who satisfied the inclusion criteria were enrolled and signed the informed consent. The patients were randomized into two groups: probiotic group (n 12) and placebo group (n 12) (Table 1). The Shapiro–Wilk test confirmed a normal distribution between the groups (*p* = 0.91, w = 0.96).

### 3.2. Gut Microbiota Analysis

At T0, gut microbiota analysis documented the absence of pathogens (Table 2).

**Figure 1:**
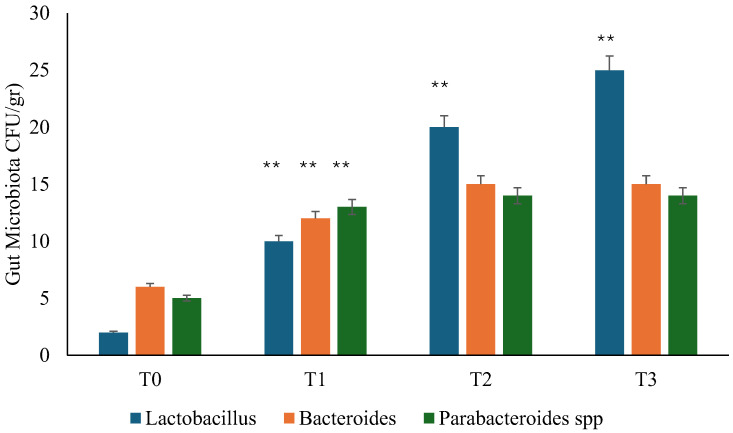
Gut microbiota analysis during the study in probiotic group. T0: admission; T1: 1 month from T0; T2: 3 months from T0; T3: 6 months from T0. ** *p* < 0.01 increase (T1 vs. T0; T2 vs. T1; T3 vs. T2).

**Figure 2:**
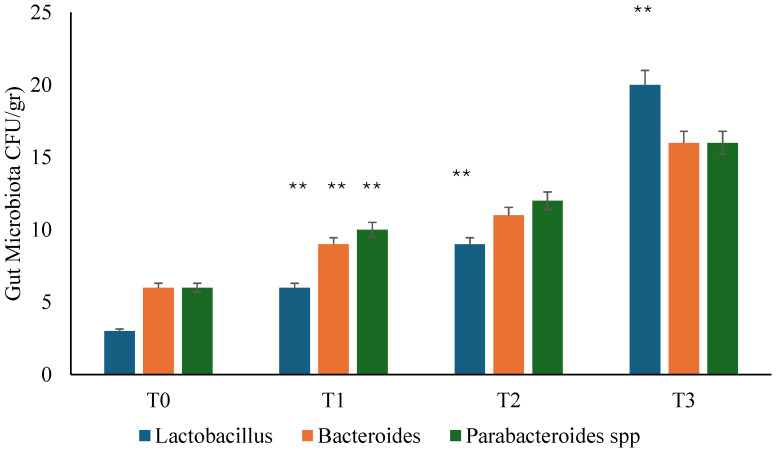
Gut microbiota analysis during follow-ups in placebo group. T0: admission; T1: 1 month from T0; T2: 3 months from T0; T3: 6 months from T0. ** *p* < 0.01 increase (T1 vs. T0; T2 vs. T1; T3 vs. T2).

Microbiological culture documented that Bacteroides, Parabacteroides, and *Lactobacillus* GG were absent or of negligible quantity at admission (T0), testifying to the presence of dysbiosis (Figure 1). During the follow-up (T1), the reappearance of Bacteroides, Parabacteroides, and *Lactobacillus* GG was recorded. In contrast, in the placebo group, it was necessary to wait 90 days (T2) to detect the development of Lactobacilli. At T3, the gut microbiota revealed a normalization of the bacterial population in all participants of both groups, but with a higher presence of Lactobacilli in the probiotic group (Figure 1 and Figure 2).

### 3.3. Seminal Microbiota Analysis

**Figure 3:**
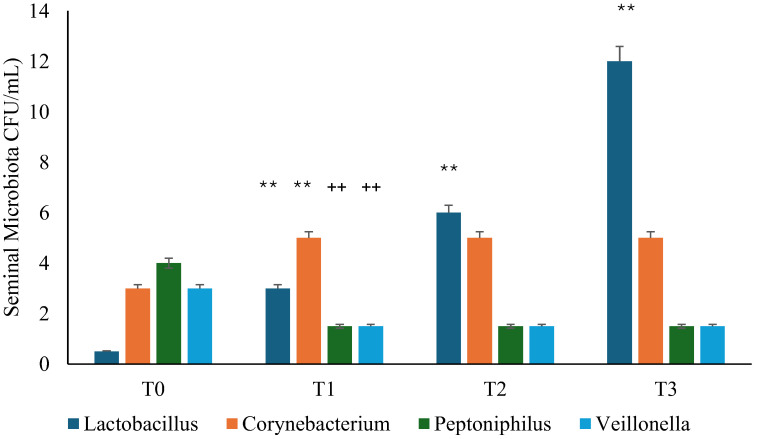
Seminal microbiota analysis was performed during follow-up in the probiotic group. T0: admission; T1: 1 month from T0; T2: 3 months from T0; T3: 6 months from T0. ** *p* < 0.01 increase (T1 vs. T0; T2 vs. T1; T3 vs. T2); ++ *p* < 0.01decrease (T1 vs. T0).

**Figure 4:**
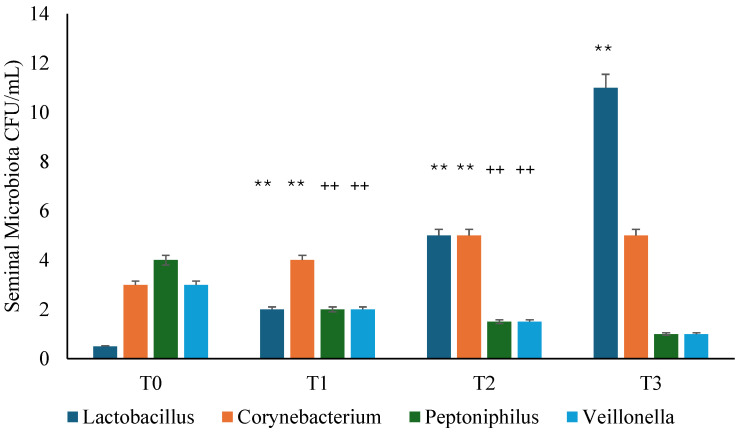
Seminal microbiota analysis during the study in placebo group. T0: admission; T1: 1 month from T0; T2: 3 months from T0; T3: 6 months from T0. ** *p* < 0.01 increase (T1 vs. T0; T2 vs. T1; T3 vs. T2); ++ *p* < 0.01 decrease (T1 vs. T0; T2 vs. T1).

In the study, the presence of many bacterial species in the seminal fluid was evaluated and, at T0, it was found that all patients with chronic infectious prostatitis, following antibiotic treatment with ciprofloxacin, had a microbial population characterized mainly by *Corynebacterium, Peptoniphilus,* and *Veillonella* (Figure 3 and Figure 4). During the follow-up visits, the seminal fluid was re-evaluated, and we documented a normalization of the microbiota in the probiotic and placebo groups, with the appearance of Lactobacilli after 30 days (T1), though this was most significant in the probiotic group (*p* < 0.01) (Figure 3 and Figure 4).

### 3.4. Questionnaire Analysis

**Figure 5:**
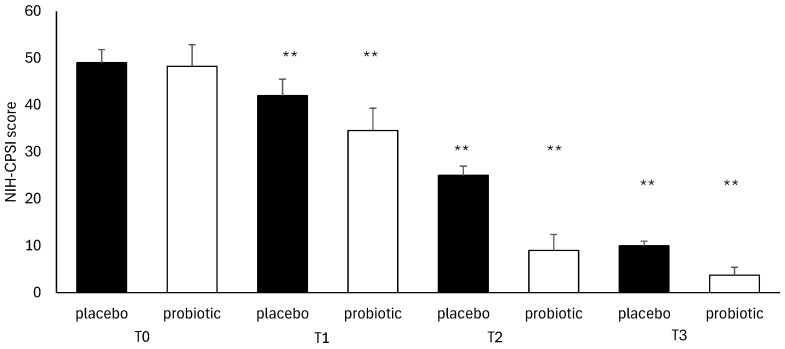
NIH-CPSI analysis during follow-ups in the placebo and probiotic groups. Data are expressed as mean ± standard deviation. T0: admission; T1: 30 days after T0, T2: 90 days after T0; T3: end of the study at 180 days after T0 ** *p* < 0.01 vs. T0.

**Figure 6:**
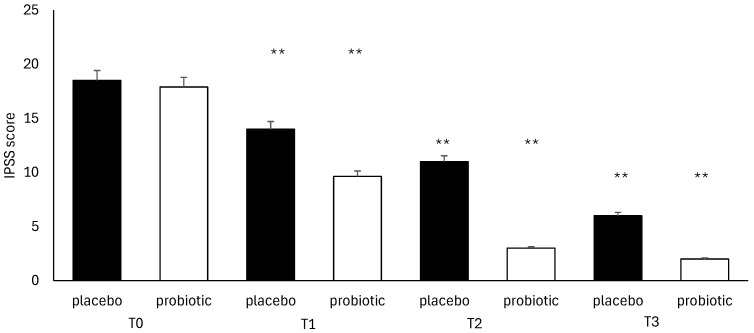
IPSS analysis during follow-ups in the placebo and probiotic groups. T0: admission; T1: 30 days after T0, T2: 90 days after T0; T3: end of the study at 180 days after T0. Data are expressed as mean ± standard deviation. ** *p* < 0.01 vs. T0.

Regarding the severity of symptoms and disease recurrence, the NIH-CPSI questionnaire was completed by all participants. It showed a non-significant difference between the placebo group and the probiotic group from T0 (*p* = 0.00) to T3 (*p* = 0.187), with a comparable decrease between the two groups during the treatment (Figure 5). The IPSS test used to evaluate urinary symptoms also produced a similar result. In this case, the difference was not significant (pT3 = 0.546) (Figure 6).

All participants completed the IIEF-5 questionnaire. The total IIEF-5 score ranged between 0 and 25. In the probiotic group compared to the placebo group, we documented an improvement in erective function, as shown in Table 3.

### 3.5. Clinical Evaluation

**Figure 7:**
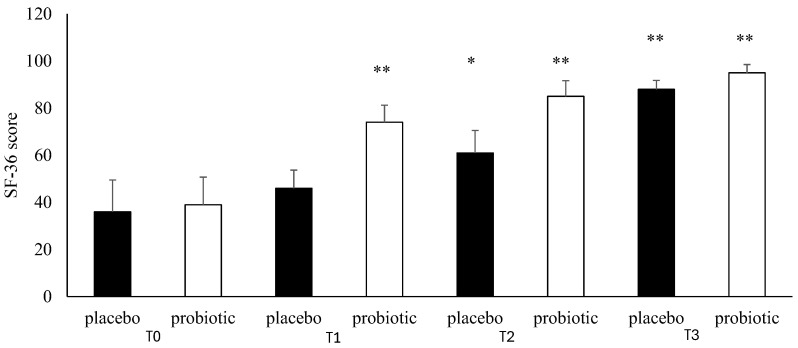
SF-36 analysis during follow-ups in the placebo and probiotic groups. T0: admission; T1: 30 days after T0, T2: 90 days after T0; T3: end of the study at 180 days after T0. Data are expressed as mean ± standard deviation. * *p* < 0.05 vs. T0; ** *p* < 0.01 vs. T0.

**Figure 8:**
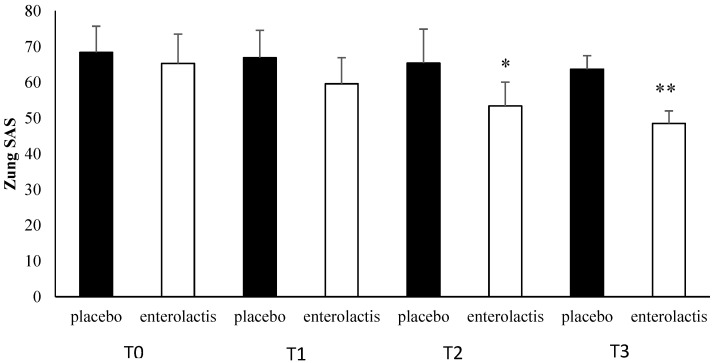
Zung SAS analysis during follow-ups in placebo and probiotic groups. T0: admission; T1: 30 days after T0, T2: 90 days after T0; T3: end of the study at 180 days after T0. Data are expressed as mean ± standard deviation. * *p* < 0.05 ** *p* < 0.01.

**Figure 9:**
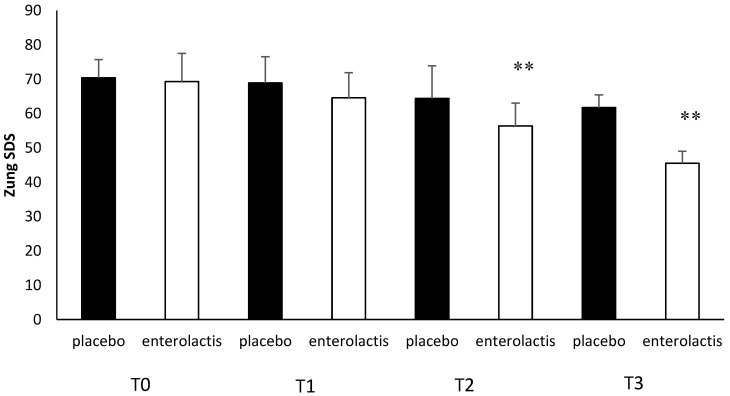
Zung SDS analysis during follow-ups in the placebo and probiotic groups. T0: admission; T1: 30 days after T0, T2: 90 days after T0; T3: end of the study at 180 days after T0. Data are expressed as mean ± standard deviation. ** *p* < 0.01 vs. T0.

Patients enrolled in the L. Casei DG^®^ group documented a significantly faster recovery (*p* < 0.01) compared to placebo, with a decrease in the duration of disease relapse and the appearance of milder symptoms with a remarkable improvement in both quality of life and mood. The SF-36 questionnaire score showed a time-related significant difference (*p* < 0.01) between the two groups studied (Figure 7). In T1, we recorded a significant improvement (*p* < 0.01) in the SF-36 score vs. T0 with respect to the placebo group. The same data were recorded in T2 and T3 (*p* < 0.01) (Figure 7). In addition, comparing the mean Zung SDS and Zung SAS between the placebo and control groups, we observed, at T3, a higher score in the placebo group compared to T0 with the difference between the groups being significant (*p* < 0.01) (Figure 8 and Figure 9).

## 4. Discussion

### 4.1. Main Findings

Here, we evaluated the effect of probiotics as an add-on treatment in patients with clinical recurrences of CBP, through gut microbiota modification analysis. We demonstrated, by using a randomized controlled study, that compared to patients who took only placebo following antibiotic therapy, participants who took the probiotic containing *Lactobacillus casei* DG^®^ achieved milder symptoms and a faster recovery from prostatitis relapses.

### 4.2. Results in the Context of Previous Studies

As stated before, recently, interest has increased among researchers for the use of an antibiotic-sparing approach in the management of CBP, in particular, for the use of probiotics. This is due to the fact that CBP requires the use of long-lasting treatments (fluoroquinolones up to 6 months, depending on the severity of the pathological form) and which, therefore, increase the risk of developing antibiotic resistance and triggering interactions, especially in poly-treated patients [37]. As pointed out before, probiotics belong to strains already present in the human intestine and which, once they reach the enteric tract, multiply and rebalance the intestinal microflora. Therefore, probiotics must resist the digestive action of gastric juice, bile salts, and digestive enzymes and, once they reach the intestine, must be able to adhere to intestinal cells, multiply, and colonize it. Therefore, there must be a beneficial effect by antagonizing pathogenic microorganisms without, however, triggering immune reactions. Probiotics are commonly used to strengthen intestinal immunity and to counteract enteritis and diarrhea that is a result of infection, is drug-related or results from food intolerance. Currently, however, many studies are underway to find new possible therapeutic uses given the varied influence that microbiota regulation exerts on many systems. The gut microbiota, in fact, is considered almost as a “forgotten organ”, that is capable of establishing multidirectional communications with other organs [38]. Many studies have shown that probiotics promote the development of the postnatal immune system, inhibiting activated CD4+ T cells [39]. In particular, lactobacilli promote the activation of dendritic cells, which are essential for activating the immune response [40]. In our study, we evaluated the effects of a formulation containing 24 billion live cells of *Lactobacillus casei* DG^®^ in managing chronic bacterial prostatitis. *Lactobacillus casei* DG^®^ is a Gram-positive bacterium normally present in our gut flora and commonly used to modulate the structure and functionality of the gut microbiota [19,41,42]. All enrolled patients completed the study and showed a normalization in the composition of gut and seminal microorganisms at the end of the treatment. However, patients enrolled in the *Lactobacillus casei* DG^®^ group presented a faster recovery from prostatitis and milder symptoms than patients who took the placebo alone. It is probably the reason for a faster clinical recovery of normal gut microflora demonstrated also by other authors [19,43]. The use of *Lactobacillus casei* DG^®^ has been shown to result in a quicker clinical recovery of the normal gut microflora. This reduces the risk of bacterial transmigration to the prostate tissue by rapidly lowering the inflammatory status of the gut mucosa and its permeability. In this regard, *Lactobacillus casei* DG^®^ may be a promising antibiotic-sparing strategy for averting CBP symptoms. Additionally, patients in the *Lactobacillus casei* DG^®^ group reported a significantly quicker recovery than those in the placebo group, as evidenced by a reduction in the length of time it took for the condition to recur, the onset of less severe symptoms, and an increase in quality of life (SF-36 questionnaire—*p* < 0.01). Additionally, the mean Zung SDS and Zung SAS questionnaire scores differed between the two groups (*p* < 0.01), suggesting that *Lactobacillus casei* DG^®^ may have a function in regulating the gut–brain axis and lessening the psychological effects of CBP [44]. Finally, in enrolled patients, we did not record the development of adverse drug reactions or drug interactions, indicating that the probiotics are safe in all patients.

## 5. Conclusions

Our data suggest that in patients with chronic prostatitis, the use of *Lactobacillus casei* DG^®^ after an antimicrobial treatment is safe and effective in the improvement of the days free of symptoms and is also able to improve the quality of life through an early restoration of the gut microbiota.

## 6. Limitations, Strengths, and Future Directions

The major limitation of this study is the low number of enrolled patients; therefore, a large study could be performed to validate our data. In particular, further studies could provide evidence that probiotic use in the therapeutic response to chronic bacterial prostatitis can limit the need for long-term use of antimicrobials, thereby reducing the risk of adverse drug reactions, drug interactions, and antimicrobial resistance development.

## Figures and Tables

**Table 1:** Demographics and clinical characteristics of the 24 enrolled patients. Data are expressed as mean ± standard deviation or as number (percentages).

Characteristics	Data	Probiotic Group	Placebo Group
Age	50 ± 3.1	50.4 ± 2.5	49.7 ± 3.4
Body Mass Index (Kg/m^2^)	27.7 ± 2.7	28.0 ± 2.5	27.4 ± 2.7
Occupation Status			
Sedentary	11	4	7
Manual	13	8	5
Comorbidity			
Copd	3	2	1
Diabetes Mellitus Type 2	6	3	3
Hypertension	8	1	7
Dyslipidemia	4	4	0
Metabolic Syndrome	3	2	1

Of the 24 patients with chronic bacterial prostatitis who participated in the study, all completed the course of treatment.

**Table 2:** QIAstat-Dx^®^ Gastrointestinal Panel evaluation. Data are expressed as total cycle for each run. Data lower than 29 represent positive values; data from 32 to 39 require a clinical evaluation. T0: admission; T1: 30 days after T0, T2: 90 days after T0; T3: end of the study at 180 days after T0.

Bacteria	T0	T1	T2	T3
*Escherichia coli*	36	34	38	36
*C. difficile*	51	49	52	54
*Campylobacter* spp. (*C. jejuni*, *C. upsaliensis*, and *C. coli*)	59	53	54	56
*Clostridioides difficile tcdA/tcdB*	51	52	51	54
Enteroaggregative *E. coli*	56	58	59	59
Enteropathogenic *E. coli*	57	58	59	58
Enterotoxigenic *E. coli eltA/estA*	61	62	59	62
Shiga toxin–producing *E. coli stx1/stx2*	59	62	61	58
Shiga toxin–producing *E. coli stx1/stx2 O157*	56	58	59	61
Enteroinvasive *E. coli*/Shigella (*S. sonnei*, *S. fexneri*, *S. boydii*, and *S. dysenteriae*)	61	62	64	62
*Plesiomonas shigelloides*	59	60	58	59
*Salmonella* spp.	63	64	61	62

**Table 3:** IIEF-5 score achieved at admission (T0) and at T3 in the placebo and probiotic groups (normal range 0-25). T0: admission; T3: end of the study at 180 days after T0.

Symptoms				
IIEF-5 (Range: 0–25 Points)	Groups at T0		Groups at T3	
	Probiotic	Placebo	Probiotic	Placebo
Normal (IIEF-5 22–25 points)	1	2	4	2
Mild (IIEF-5 17–21 points)	5	5	7	6
Mild to moderate (IIEF 12–16 points)	3	1	1	2
Moderate to severe (IIEF 8–11 points)	1	2	0	1
Severe (IIEF-5 0–7 points)	2	2	0	1

## Data Availability

Data are unavailable due to privacy or ethical restrictions in accordance with Italian bylaw.
